# Early prediction of median survival among a large AIDS surveillance cohort

**DOI:** 10.1186/1471-2458-7-127

**Published:** 2007-06-27

**Authors:** Wayne TA Enanoria, Alan E Hubbard, Mark J van der Laan, Mi Chen, Juan Ruiz, John M Colford

**Affiliations:** 1 Division of Epidemiology, School of Public Health, University of California at Berkeley, Berkeley, California, USA; 2 Division of Biostatistics, School of Public Health, University of California at Berkeley, Berkeley, California, USA; 3 Centers for Disease Control and Prevention, Atlanta, Georgia, USA; 4 California Department of Health Services, Office of AIDS, Sacramento, California, USA

## Abstract

**Background:**

For individuals with AIDS, data exist relatively soon after diagnosis to allow estimation of "early" survival quantiles (*e.g.*, the 0.10, 0.15, 0.20 and 0.30 quantiles, etc.). Many years of additional observation must elapse before median survival, a summary measure of survival, can be estimated accurately. In this study, a new approach to predict AIDS median survival is presented and its accuracy tested using AIDS surveillance data.

**Methods:**

The data consisted of 96,373 individuals who were reported to the HIV/AIDS Reporting System of the California Department of Health Services Office of AIDS as of December 31, 1996. We defined cohorts based on quarter year of diagnosis (*e.g.*, the "931" cohort consists of individuals diagnosed with AIDS in the first quarter of 1993). We used early quantiles (estimated using the Inverse Probability of Censoring Weighted estimator) of the survival distribution to estimate median survival by assuming a linear relationship between the earlier quantiles and median survival. From this model, median survival was predicted for cohorts for which a median could not be estimated empirically from the available data. This prediction was compared with the actual medians observed when using updated survival data reported at least five years later.

**Results:**

Using the 0.15 quantile as the predictor and the data available as of December 31, 1996, we were able to predict the median survival of four cohorts (933, 934, 941, and 942) to be 34, 34, 31, and 29 months. Without this approach, there were insufficient data with which to make any estimate of median survival. The actual median survival of these four cohorts (using data as of December 31, 2001) was found to be 32, 40, 46, and 80 months, suggesting that the accuracy for this approach requires a minimum of three years to elapse from diagnosis to the time an accurate prediction can be made.

**Conclusion:**

The results of this study suggest that early and accurate prediction of median survival time after AIDS diagnosis may be possible using early quantiles of the survival distribution. The methodology did not seem to work well during a period of significant change in survival as observed with highly active antiretroviral treatment, but results suggest that it may work well in a time of more gradual improvement in survival.

## Background

Since the beginning of the AIDS epidemic, the prediction of trends in survival after an AIDS diagnosis has been important for planning health care services and for monitoring the impact of the epidemic. Temporal associations between improved survival following the introduction of expanded treatment options provide population-based evidence that there may be beneficial treatment effects long before these hypotheses can be tested formally. In a time when health care resources are limited and health priorities must be established, it is crucial to project the short-term mortality after AIDS for future planning of health care resources [1].

Temporal trends and improvements in survival with AIDS were reported early in the epidemic even before the introduction of advances in therapy [2]. Shortly after the introduction of zidovudine therapy, temporal trends in survival were (eventually) noted using surveillance data [3]. Other registry-based studies investigated the relationship between survival following an AIDS diagnosis and calendar date of diagnosis [4,5]. These studies consistently showed marked improvements in AIDS survival after the introduction of zidovudine therapy and *Pneumocystis carinii *pneumonia prophylaxis. More recently, the introduction of highly active antiretroviral therapy (HAART) has renewed the idea of examining trends in survival after an AIDS diagnosis in order to study both the short- and long-term effects of these new drugs on HIV-related morbidity and mortality.

In this study, a new approach was implemented to predict AIDS survival and test its accuracy using AIDS surveillance data. The purpose of this study was: (1) to determine the earliest quantile (such as 0.10, 0.15, or 0.20) of the survival distribution that can be used to predict accurately a cohort's subsequently observed median survival, and (2) to estimate the survival quantiles using the Inverse Probability of Censoring Weighted (IPCW) estimator [6] in order to improve the prediction methodology in the common situation with registry data in which death is subject to delays in reporting [7].

For cohorts of individuals who have recently been diagnosed with AIDS, data exist for "early" survival quantiles (such as the 0.10, 0.15, 0.20 quantiles, etc.) but many years of additional observation must elapse before later quantiles, such as the median (0.50 quantile) can be estimated with accuracy. Assuming a linear relationship between the early survival quantile and the median survival, an early prediction of the median value for a cohort's eventual survival distribution is compared to the actual or true median value for the cohort. If the predicted median is accurate, then early estimation of AIDS survival is possible and will be of great benefit to health care planners developing strategies and financing for the health care needs of these patients. Additionally, such an accurate, early prediction methodology could be extended to other large, population-based surveillance systems where survival prediction is a major goal.

## Methods

### California AIDS surveillance data

The California Department of Health Services, Office of AIDS (OA), in cooperation with the Centers for Disease Control and Prevention (CDC), maintains a registry of all reports of AIDS cases in California. This registry, the HIV/AIDS Reporting System (HARS), contains demographic, risk factor and limited clinical information on each reported case. A HARS data set as of December 31, 2001 was used to obtain four variables: the dates of AIDS diagnosis (month and year only; the day was assumed to be 15 in order to calculate a date), the dates of death (if reported), the dates the deaths were reported to the CDC, and the date each case was entered into the registry. Dates of death are updated periodically by local city and county health departments and by OA using the California Death Registry and the National Death Index.

The State of California Health and Human Services Agency Committee for the Protection of Human Subjects and the Committee for the Protection of Human Subjects at the University of California at Berkeley approved the use of these data for this purpose.

### Identification of cohorts of AIDS patients as of December 31, 1996

The date of AIDS diagnosis is the date of the first condition that would allow a person to be classified as having AIDS under the 1993 change in the AIDS case definition [8]. This definition was retroactively applied to cases diagnosed prior to 1993. Cases were grouped into cohorts defined by the calendar quarter of their AIDS diagnosis. For example, a person diagnosed in November of 1992 (*i.e.*, the fourth quarter of 1992) was classified into the "924" cohort. All AIDS cases diagnosed according to the 1993 change in the AIDS case definition and entered into the HARS Registry between January 1, 1983 and December 31, 1996 were eligible to be included in the study.

### Determination of survival from AIDS diagnosis to death

CDC receives information from California's HIV/AIDS Registry on a monthly basis. For all newly-reported deaths, the date on which the death was first reported to the CDC is recorded. In order to re-create the death information that would have been available to any investigator as of December 31, 1996, death dates were included only if they were reported on or before this date. Survival time was defined as the time elapsed from the date of the AIDS diagnosis until death from all causes, or until December 31, 1996, the date of analysis for the study.

### The Inverse Probability of Censoring Weighted estimator

For many sources of registry-based data, there is a delay between the recording of vital status and its availability for analysis. In such situations, the analyst may assume mistakenly that those who are not yet known to have died are still alive when, in fact, some of these individuals may have died but the deaths have not yet been reported to the registry. The use of the Kaplan-Meier (K-M) estimator to estimate survival in this situation has been shown to be inconsistent and to yield biased results [9]. Following the approach of Robins and Rotnitzky [10], van der Laan and Hubbard [6] and Hubbard *et al. *[11] proposed a simple inverse probability of censoring weighted estimator to account for this delay in vital status information and this estimator was applied in this study.

The study sample consists of 56 cohorts of individuals with AIDS defined by the quarter year of diagnosis. Since the censoring date (the date of analysis) is December 31, 1996, individuals diagnosed with AIDS in the 951 cohort who survived the entire period can only have censoring times equal to 23 months (for those diagnosed in January), 22 months (for those diagnosed in February), or 21 months (for those diagnosed in March). One possible concern with using the IPCW estimator to estimate the survival distribution is that the estimator may perform poorly if the censoring distribution has all of its weight on a small set of times, as observed with this data. If there are subjects for whom the reporting time is greater than the support of possible censoring times, the IPCW may be biased [11]. In order to account for this, artificial censoring was used to augment the estimator.

Each case was assigned a new, uniformly distributed censoring time from 0 months to the maximum censoring time according to the cohort to which each case belonged. For example, the individuals diagnosed in the first quarter of 1995, the 951 cohort, were each assigned randomly a censoring time from a uniform distribution ranging from 0 months to 23 months, the maximum censoring time for this cohort. Similarly, the members of the 941 cohort were each assigned a censoring time from a uniform distribution from 0 months to 35 months, the members of the 931 cohort from 0 months to 47 months, and so forth. The censoring time for each individual was taken to be the minimum of this new censoring time or the original censoring time defined as the time elapsed from their date of diagnosis to December 31, 1996. By doing so, an artificial censoring distribution is created with more uniform mass over the possible times of death for each of the cohorts.

The reason to artificially censor the date arises from the type of censoring distribution encountered in these data. Specifically, subjects are enrolled within a narrow window of time (three months) for each cohort and all subjects are censored at the same chronological time. Thus, the censoring distribution has all of its mass over a three month period. The consequence of this is the potentially high variability in the IPCW estimator for quantiles within the support of censoring. By artificially censoring the data, censoring is "spread" over a larger interval which reduces the variability of estimates of survival at later quantiles. The cost is that the variability of survival estimates of earlier quantiles is increased by censoring originally uncensored observations.

### Prediction of median estimates of survival

Since our goal was to use early survival experiences to predict later survival, we examined the relation between the early quantiles (*i.e.*, 0.10, 0.15, 0.20, and 0.30 quantiles) of the survival distribution and the 0.50 quantile. Assuming a linear relationship, predicted median estimates were calculated based on the estimation of the linear model by entering the observed early quantile into the model. By assuming a linear model, this implies that our method only works so long as there is the same systematic shift in the survival distribution over time. That is, if the early quantile increases over time for a particular cohort, our method works only if the later quantile increases as well.

### The "true" quantiles of the survival distribution

The "true" quantiles (*i.e.*, the best possible estimate of the quantiles) of the survival distribution were assumed to be the quantiles of survival estimated empirically from the data using the IPCW estimator as of December 31, 2001. This provided at least an additional five years of observation after the date of analysis upon which the early predictions were made. In order to assess the performance of the prediction method, the predicted median estimates using our method were compared to these "true" medians (*i.e.*, observed median estimates using data as of December 31, 2001) for the study sample.

## Results

### Deaths in HARS

The justification for using five years of follow-up as providing the "true" survival estimates (*i.e.*, the length of follow-up necessary for a cohort until the quantiles are "stable" and the "true" quantiles are achieved for a particular cohort diagnosed with AIDS) is based upon empirical data. Using the raw data as of December 31, 2001 (with no artificial censoring imposed), the cumulative numbers of deaths over ten years of follow-up for four cohorts were determined (Table 1). Among the deaths that were known to occur after ten years of follow up for the cohort of individuals diagnosed in 854 (n = 309 deaths reported as of December 31, 1995 among n = 317 individuals identified as part of this cohort), 83.6% of the cohort were known to have died within four years and 88.3% were reported within 5 years. On average, 80% or more of cohorts were known to have died within four years of the identification of the cohort. These results give empiric evidence that the "true" quantiles of survival are those which are observed five years after identification of the cohort and were our basis for our decision to derive the "true" estimates using data from December 2001.

**Table 1:** Cumulative number of deaths over a ten-year follow-up period for the 854, 874, 894, and 914 cohorts.

	854 Cohort n = 317			874 Cohort n = 509			894 Cohort n = 579			914 Cohort n = 593		
Date of Analysis	Number of Deaths Reported by Date of Analysis	Cumulative Deaths	% Cohort	Number of Deaths Reported by Date of Analysis	Cumulative Deaths	% Cohort	Number of Deaths Reported by Date of Analysis	Cumulative Deaths	% Cohort	Number of Deaths Reported by Date of Analysis	Cumulative Deaths	% Cohort
12/31/1985	0	0	0.0%									
12/31/1986	0	0	0.0%									
12/31/1987	196	196	61.8%	22	22	4.3%						
12/31/1988	55	251	79.2%	153	175	34.4%						
12/31/1989	14	265	83.6%	145	320	62.9%	31	31	5.4%			
12/31/1990	15	280	88.3%	80	400	78.6%	182	213	36.8%			
12/31/1991	20	300	94.6%	36	436	85.7%	143	356	61.5%	20	20	3.4%
12/31/1992	6	306	96.5%	24	460	90.4%	79	435	75.1%	203	223	37.6%
12/31/1993	0	306	96.5%	14	474	93.1%	48	483	83.4%	131	354	59.7%
12/31/1994	1	307	96.8%	8	482	94.7%	36	519	89.6%	86	440	74.2%
12/31/1995	2	309	97.5%	0	482	94.7%	14	533	92.1%	48	488	82.3%
12/31/1996				4	486	95.5%	9	542	93.6%	21	509	85.8%
12/31/1997				1	487	95.7%	3	545	94.1%	6	515	86.8%
12/31/1998							2	547	94.5%	3	518	87.4%
12/31/1999							0	547	94.5%	2	520	87.7%
12/31/2000										3	523	88.2%
12/31/2001										0	523	88.2%

### Study sample using data as of December 31, 1996

There were 96,754 AIDS cases diagnosed between 1978 through 1996 and entered into the HARS database on or before December 31, 1996. Of those, 84 cases (0.1%) were excluded who were reported as having negative survival times (n = 1) or negative reporting times (n = 83). After excluding 297 cases (0.31%) diagnosed prior to January 1, 1983 due to small sample sizes for each of these cohorts, 96,373 (99.6%) of all AIDS cases diagnosed and entered by December 31, 1996 were included in the analysis.

### Survival quantiles according to the Inverse Probability of Censoring Weighted estimator

**Figure 1:**
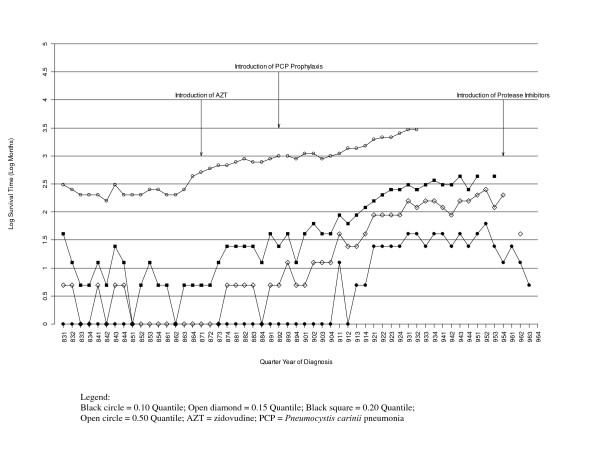
**Observed Quantiles of the Survival Distribution, 1983–1996 (HARS Data as of December 31, 1996)**.

Figure 1 shows the 0.10, 0.15, 0.20, and the 0.50 quantiles for cohorts estimated using the database as it would have existed on December 31, 1996 (plotted on a log scale). The median estimate appears to be increasing beginning with the 864 cohort and again with the 904 cohort. There are 11 cohorts (933 through 954 and 962) for which the 0.15 quantile could be estimated and the 0.50 quantile could not be estimated using the IPCW estimator.

### Predicted median estimates based on the relation of the observed median estimates and the observed 0.15 quantiles

**Figure 2:**
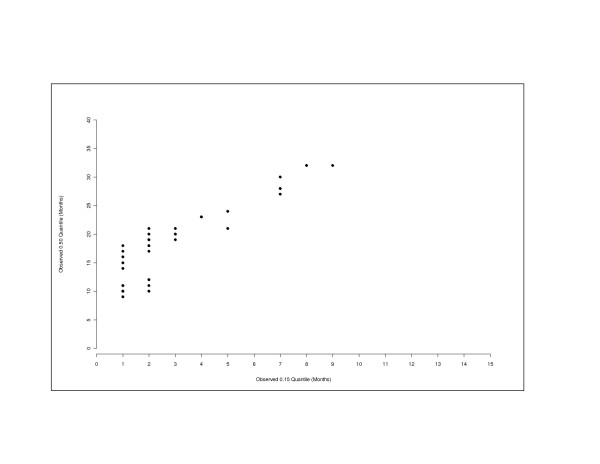
Scatterplot of the 0.15 Quantile and the Observed 0.50 Quantile as of December 31, 1996.

Table 2 shows the observed 0.15 quantile (column A) and the observed 0.50 quantile (column B) according to the IPCW estimator as of December 31, 1996. From the results of the linear regression analysis, a median could be predicted for four cohorts (cohorts 933 through 942) for which no median estimates had been observed as of December 31, 1996 (column C) and compared to the true median estimates five years later (column D). For the 933 cohort, the predicted median estimate was 34 months based on the observed 0.15 quantile (the true median based on the 2001 data was 32 months). For the 934 cohort, the predicted median estimate was 34 months (the true median based on the 2001 data was 40 months). The differences between the predicted median and the "true" median increase as the cohorts get closer to the censoring date, *i.e.*, for cohorts 934, 941, and 942. A scatterplot of the observed 0.15 and 0.50 quantiles is given in Figure 2.

**Table 2:** Predicted versus True 0.50 Quantiles using the 0.15 Quantile as a Predictor Variable.

	Data as of December 31, 1996					Data as of December 31, 2001			
		Observed Quantiles							
Cohort	n	largest follow-up time (months)	0.15 (A)	0.50 (B)	Predicted 0.50 Quantile† (C)	largest follow-up time (months)	"True" 0.15 Quantile§ (D)	True 0.50 Quantile‡ (E)	Difference (C – E)

831	140	167	2	12	16	227	2	12	4
832	154	164	2	11	16	224	1	11	5
833	204	161	1	10	13	221	1	10	3
834	205	158	1	10	13	218	1	11	2
841	277	155	2	10	16	215	1	10	6
842	315	152	1	9	13	212	1	11	2
843	397	149	2	12	16	209	1	11	5
844	418	146	2	10	16	206	1	9	7
851	531	143	1	10	13	203	1	10	3
852	624	140	1	10	13	200	1	10	3
853	718	137	1	11	13	197	1	10	3
854	689	134	1	11	13	194	1	10	3
861	876	131	1	10	13	191	1	10	3
862	955	128	1	10	13	188	1	10	3
863	1113	125	1	11	13	185	1	11	2
864	1110	122	1	14	13	182	1	15	-2
871	1316	119	1	15	13	179	1	14	-1
872	1474	116	1	16	13	176	1	15	-2
873	1484	113	1	17	13	173	2	17	-4
874	1425	110	2	17	16	170	2	17	-1
881	1649	107	2	18	16	167	2	18	-2
882	1658	104	2	19	16	164	3	19	-3
883	1759	101	2	18	16	161	2	18	-2
884	1693	98	1	18	13	158	1	18	-5
891	1930	95	2	19	16	155	2	18	-2
892	2187	92	2	20	16	152	2	19	-3
893	2047	89	3	20	18	149	3	21	-3
894	1997	86	2	19	16	146	2	18	-2
901	2265	83	2	21	16	143	3	22	-6
902	2243	80	3	21	18	140	4	22	-4
903	2285	77	3	19	18	137	3	20	-2
904	2139	74	3	20	18	134	3	20	-2
911	2611	71	5	21	23	131	5	22	1
912	2543	68	4	23	21	128	4	23	-2
913	2845	65	4	23	21	125	4	23	-2
914	2930	62	5	24	23	122	5	24	-1
921	3227	59	7	27	29	119	8	27	2
922	2920	56	7	28	29	116	7	28	1
923	2971	53	7	28	29	113	7	27	2
924	3061	50	7	30	29	110	7	29	0
931	3264	47	9	32	34	107	9	31	3
932	2903	44	8	32	31	104	8	32	-1
933	2788	41	9	*	34	101	9	32	2
934	2624	38	9	*	34	98	9	40	-6
941	2766	35	8	*	31	95	9	46	-15
942	2518	32	7	*	29	92	8	80	-51
943	2335	29	9	*	34	89	9	*	
944	2203	26	9	*	34	86	8	*	
951	2451	23	10	*	37	83	9	*	
952	2245	20	11	*	39	80	10	*	
953	1949	17	8	*	31	77	9	*	
954	1860	14	10	*	37	74	9	*	
961	1894	11	*	*	*	71	12	*	
962	1488	8	5	*	23	68	16	*	
963	1225	5	*	*	*	65	16	*	
964	475	2	*	*	*	62	9	*	

* denotes that the observed quantile could not be estimated from the data.

† The predicted 0.50 quantile is based on HARS data as of December 31, 1996.

§The true 0.15 quantile is based on HARS data as of December 31, 2001.

‡ The true 0.50 quantile is based on HARS data as of December 31, 2001.

**Figure 3:**
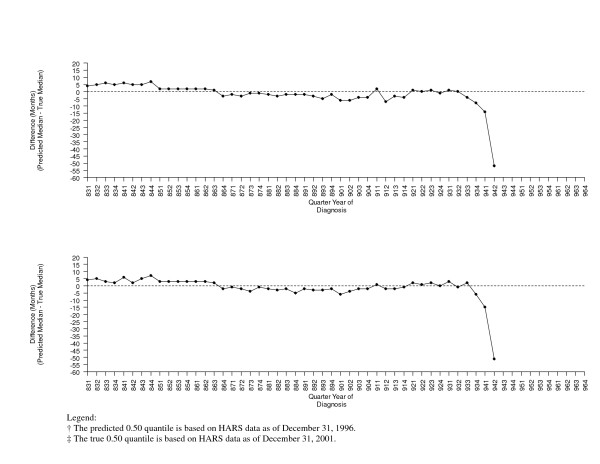
**Comparison of the Predicted† and True‡ 0.50 Quantiles using the 0.10 (top) and the 0.15 (bottom) Quantiles as Predictors**.

**Figure 4:**
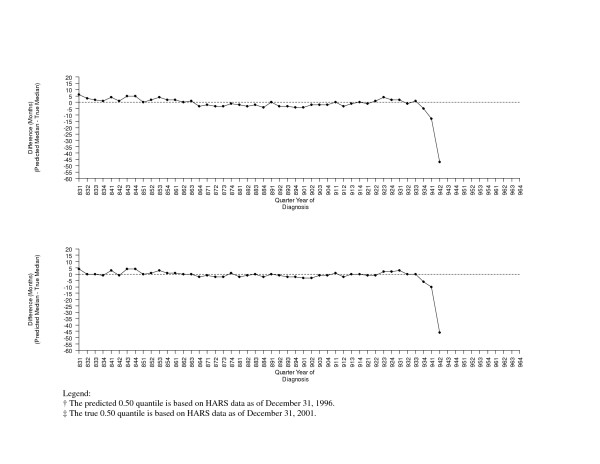
**Comparison of the Predicted† and True‡ 0.50 Quantiles using the 0.20 (top) and the 0.30 (bottom) Quantiles as Predictors**.

A comparison of the predicted medians and the true medians using other early quantiles of the survival distribution as predictors in separate linear regression models are shown graphically in Figure 3 (using the 0.10 and 0.15 quantiles as predictors) and Figure 4 (using the 0.20 and 0.30 quantiles as predictors).

A closer look at the predicted median survival estimates according to the IPCW estimator (Table 2) shows that this technique overestimated the median for earlier cohorts (suggesting a steeper linear slope) and underestimated the median for later cohorts (suggesting a less steep slope). This would suggest that the relation between the 0.15 quantile and the 0.50 quantile while assumed to be linear is changing over time. Thus, predicted median survival estimates based on a model with an interaction between the 0.15 quantile and calendar time was evaluated (Table 3). The inclusion of an interaction term with time yielded a predicted median that was closer to the truth (in comparison to the model without an interaction term) for three cohorts for which a median could not be observed at the time the prediction was made (933, 941, and 942).

**Table 3:** Predicted versus True 0.50 Quantiles using the 0.15 Quantile as a Predictor Variable based on a linear model with an interaction term.

	Data as of December 31, 1996					Data as of December 31, 2001			
			Observed Quantiles						
Cohort	n	largest follow-up time (months)	0.15 (A)	0.50 (B)	Predicted 0.50 Quantile† (C)	largest follow-up time (months)	"True" 0.15 Quantile§ (D)	True 0.50 Quantile‡ (E)	Difference (C – E)

831	140	167	2	12	9	227	2	12	-3
832	154	164	2	11	10	224	1	11	-1
833	204	161	1	10	9	221	1	10	-1
834	205	158	1	10	9	218	1	11	-2
841	277	155	2	10	11	215	1	10	1
842	315	152	1	9	10	212	1	11	-1
843	397	149	2	12	12	209	1	11	1
844	418	146	2	10	12	206	1	9	3
851	531	143	1	10	11	203	1	10	1
852	624	140	1	10	11	200	1	10	1
853	718	137	1	11	12	197	1	10	2
854	689	134	1	11	12	194	1	10	2
861	876	131	1	10	13	191	1	10	3
862	955	128	1	10	13	188	1	10	3
863	1113	125	1	11	13	185	1	11	2
864	1110	122	1	14	14	182	1	15	-1
871	1316	119	1	15	14	179	1	14	0
872	1474	116	1	16	14	176	1	15	-1
873	1484	113	1	17	15	173	2	17	-2
874	1425	110	2	17	16	170	2	17	-1
881	1649	107	2	18	17	167	2	18	-1
882	1658	104	2	19	17	164	3	19	-2
883	1759	101	2	18	17	161	2	18	-1
884	1693	98	1	18	16	158	1	18	-2
891	1930	95	2	19	18	155	2	18	0
892	2187	92	2	20	18	152	2	19	-1
893	2047	89	3	20	20	149	3	21	-1
894	1997	86	2	19	19	146	2	18	1
901	2265	83	2	21	19	143	3	22	-3
902	2243	80	3	21	21	140	4	22	-1
903	2285	77	3	19	21	137	3	20	1
904	2139	74	3	20	22	134	3	20	2
911	2611	71	5	21	24	131	5	22	2
912	2543	68	4	23	23	128	4	23	0
913	2845	65	4	23	24	125	4	23	1
914	2930	62	5	24	25	122	5	24	1
921	3227	59	7	27	28	119	8	27	1
922	2920	56	7	28	28	116	7	28	0
923	2971	53	7	28	28	113	7	27	1
924	3061	50	7	30	29	110	7	29	0
931	3264	47	9	32	31	107	9	31	0
932	2903	44	8	32	31	104	8	32	-1
933	2788	41	9	*	32	101	9	32	0
934	2624	38	9	*	32	98	9	40	-8
941	2766	35	8	*	32	95	9	46	-14
942	2518	32	7	*	31	92	8	80	-49
943	2335	29	9	*	33	89	9	*	
944	2203	26	9	*	34	86	8	*	
951	2451	23	10	*	35	83	9	*	
952	2245	20	11	*	36	80	10	*	
953	1949	17	8	*	34	77	9	*	
954	1860	14	10	*	36	74	9	*	
961	1894	11	*	*	*	71	12	*	
962	1488	8	5	*	31	68	16	*	
963	1225	5	*	*	*	65	16	*	
964	475	2	*	*	*	62	9	*	

* denotes that the observed quantile could not be estimated from the data.

† The predicted 0.50 quantile is based on HARS data as of December 31, 1996.

§The true 0.15 quantile is based on HARS data as of December 31, 2001.

‡ The true 0.50 quantile is based on HARS data as of December 31, 2001.

### Median survival according to the Kaplan-Meier estimate of survival for four cohorts of AIDS cases

Our methodology enabled us to predict the median survival for four cohorts for which a median had not yet been observed: the 933, 934, 941, and 942 cohorts. Using our methodology, we estimated the median survival for the cohort 933 would be 34 months as of December 31, 1996 (Tables 2 and 3). Using traditional techniques (*i.e., *using the K-M estimator of survival), we would not have been able to observe a median of 34 months for this cohort until September 30, 2000 (almost four years later) (Additional File 1). Thus, our prediction method enabled us to make a prediction for cohorts almost four years earlier than it would be observed using traditional techniques. For the cohort 934, we estimated the median survival would be 34 months. As of December 31, 2001 (the closing date for our dataset), we still had not observed a median of 34 months according to the K-M estimator. For the 934 cohort, any median estimate would not be observed using traditional methods until May 31, 1998, 17 months after the prediction was made (December 31, 1996) using our methodology (Additional File 2).

### Predicted median estimates based on other dates of analyses

We also examined the performance of our prediction method using two other dates of analyses other than December 31, 1996. Prediction median estimates for data as of December 31, 1992 and December 31, 1994 are presented in Additional Files 3 and 4 respectively.

## Discussion

For cohorts of individuals who have been diagnosed recently with AIDS, data exist relatively soon after diagnosis for estimating "early" survival quantiles (such as the 0.10, 0.15, 0.20 quantiles, etc.) but many years of additional observation must elapse before later quantiles can be estimated accurately. The purpose of this study was to determine if median survival could be predicted accurately using earlier quantiles of survival distributions provided by AIDS surveillance data. Our approach for predicting median survival consisted of two components: (1) the estimation of quantiles of the survival distribution using the IPCW estimator, and (2) the use of a linear model to reflect the relationship between the early quantile and the later quantile. The utility of such an approach would allow early information to predict later unobserved survival patterns in order to accurately identify changes in population-based survival years before such changes are observed. If accurate estimation could be achieved, this approach could offer a method for researchers to estimate the expected survival distribution after AIDS diagnosis (or after many conditions for which surveillance databases are maintained such as cancer). This approach enables accurate predictions of changes in survival among HIV-infected individuals like that observed in 1987 [3-5,12] and more notably with the advent of the use of highly active antiretroviral therapies [13-16].

The class of IPCW estimators has been developed in order to improve more traditional techniques in situations where these techniques may lead to biased estimates of survival. IPCW estimators have been applied to many types of survival problems such as correcting for non-compliance and dependent censoring in the examination of a beneficial treatment effect on survival [17] and non-parametric survival estimation when death is reported with delay [11], as in this study.

In this study, the 0.15 quantile of the survival distribution predicted accurately the median survival for cohorts diagnosed before the third quarter of 1993. In addition, the 0.15 quantile of the survival distributions predicted accurately the median survival in the short-term for two cohorts (933 and 934) for which a median estimate could not be estimated at the time of analysis (December 31, 1996). By using traditional methods (*i.e., *the K-M estimator) and without the use of our methodology, at least six months of additional follow-up would be required to observe any median survival estimate for the 933 cohort and at least 45 months until the predicted median of 34 months (as predicted by our methodology) would be observed for this cohort. This demonstrated that our methodology not only provides an accurate estimate of median survival, but an estimate of median survival long before traditional approaches would allow.

The results of this study suggest that our methodology yields an accurate prediction of median survival for cohorts diagnosed at least three years earlier than the date when the prediction is made. For example, the difference between the predicted and the true median survival was ≤ 6 months for the cohorts 933 and 934 but greater than 6 months for the cohorts 941 and 941 using data as of December 31, 1996. The "true" median estimate (using data as of December 31, 2001) according to the IPCW estimator for the 942 cohort was estimated to be 80 months. This may indeed be an early estimate of the median survival for this cohort and, as more data for this cohort becomes available, this median estimate may decrease over time like that observed with the K-M estimator (Additional File 1). This early estimate would greatly affect our assessment of the accuracy of our predictions since we are using this estimate as the "true" median survival. As a result, if one applies this methodology now in the second quarter of 2006, we could only expect to be able to predict with accuracy the median survival for cohorts that were diagnosed in the second quarter of 2003 or earlier. This methodology would not appear to work for cohorts diagnosed later than the second quarter of 2003.

The ability of the method, however, to accurately predict median survival in the short-term based on historical data is greatly influenced by three factors: (1) the variability of the estimates of the various quantiles of the survival distribution, (2) the assumption that the relation between the early quantile and the later quantile (median) can be represented by a linear model, and (3) this relation will remain the same in the short-term.

The estimates of the various quantiles of the survival distribution are affected greatly by two sources of bias: delays in diagnosis and delays in death reports. The fact that patients who were in HARS as of a particular date of analysis were included in the study sample obviously excluded those who were diagnosed before this date of analysis but were reported sometime after. Delays in diagnosis affect the estimates of survival by introducing additional individuals into each of the cohorts and, depending on their individual survival times, may affect the observed quantiles of the survival distribution. It is unclear how such individuals would affect the estimates of survival, but this potential for bias was eliminated by only including those who were reported by a fixed data of analysis (*e.g.*, December 31, 1996) in order to mimic the "real world" situation in which only those patients currently entered into a database are available for analysis. This should not detract from the utility of the estimation procedure since the predicted medians were compared with additional data five years after the prediction was made using only those patients who were selected in the original study sample.

In addition, delays in the reporting of death can bias estimates of survival if one assumes that a case is alive if a death date has not been reported. In this study, the use of the IPCW estimator is an attempt to mitigate the potential for bias in estimating survival after an AIDS diagnosis due to reporting delays of death. This estimator adjusts the estimates of survival for a given cohort to account for delays in death reports, provided that the delay of death report distribution is known. The bias introduced by failing to account for delays in death reporting when estimating survival after an AIDS diagnosis has already been established [11].

For simplicity, the prediction method assumed that the early quantile such as the 0.15 quantile, a representation of the "early"' survival experience was linearly related to the median of the survival estimate, the "later survival experience". Higher-dimensional models were explored but did not improve the predictive ability (data not shown). In assuming a linear relationship and extrapolating the observed relationship to the future, an additional assumption made was that this relationship would remain as observed in the past, at least in the short-term. Validation from the more recent cohorts (*i.e.*, the 933 and 934 cohorts) confirms that the linear model accurately predicts median survival in the short-term, but may not perform well for all cohorts (*e.g.*, the 941 and 942 cohorts). Assuming that HAART first became available with the approval of Saquinavir (hard-gel) in December 1995, the 941 and 942 cohorts would have been introduced to HAART earlier after diagnosis (21 months for the 941 cohort and 18 months for the 942 cohort) in comparison to the 933 and 934 cohorts (27 months and 24 months respectively). When comparing survival across different cohorts diagnosed over time, we would expect the later cohorts to demonstrate a shift in survival, thus violating any observed linear relationships between earlier quantiles and later quantiles observed in the past.

## Conclusion

This investigation suggests that this approach to survival estimation accurately predicted subsequent survival experience observed in two of these cohorts (the 933 and 934 cohorts). It is notable that the technique did not perform well during a period of rapid increase in AIDS survival that is not unlike the presently observed increases in survival influenced by current advances in therapy. However, the performance of the methodology before the introduction of HAART suggests that this methodology may work well in a time of more gradual improvement in survival with antiretroviral treatment. This technique may also have application in other areas of research (*e.g*. cancer surveillance) where population changes in survival have been observed and should be validated using additional data.

## Competing interests

The author(s) declare that they have no competing interests.

## Authors' contributions

JC and WE conceived of the study and WE drafted the manuscript. WE conducted the analyses under the guidance of AH and MvdL who created the novel survival methodology presented in this paper. MC and JR participated in acquisition of the data and interpretation of the data analyses. All authors read and approved the final manuscript.

## Pre-publication history

The pre-publication history for this paper can be accessed here:



## Supplementary Material

Additional File 1 Median survival (months) for four cohorts of AIDS cases at different dates of analysis according to the Kaplan-Meier estimator, December 1996 – December 2001. This table gives the estimate of median survival according to the Kaplan-Meier estimator for four cohorts for different dates of analysis in order to illustrate when a median survival estimate would be observed using traditional methods. Click here for file

Additional File 2 Comparison of Prediction Methodology with Traditional Methods (Kaplan-Meier Estimator). This table gives the earliest date the true median survival estimate could be observed and estimated using traditional methods (the Kaplan-Meier estimate), as well as the date any median survival estimate could be observed and estimated using the traditional approach. Click here for file

Additional File 3 Predicted 0.50 Quantile versus "True" 0.50 Quantile according to the Inverse Probability of Censoring Weighted Estimate of Survival, 1983–1992. This table gives predicted and true 0.50 quantile estimates of survival using the methodology presented in the paper using a dataset as of December 31, 1992 and comparing it with results as of December 31, 1997 (assumed to be the "truth"). Click here for file

Additional File 4 Predicted 0.50 Quantile versus "True" 0.50 Quantile according to the Inverse Probability of Censoring Weighted Estimate of Survival, 1983–1994. This table gives predicted and true 0.50 quantile estimates of survival using the methodology presented in the paper using a dataset as of December 31, 1994 and comparing it with results as of December 31, 1999 (assumed to be the "truth"). Click here for file
