# Supplementary material for: Early prediction of median survival among a large AIDS surveillance cohort
Source: BMC Public Health. 2007 Jun 27;7:127. doi: 10.1186/1471-2458-7-127 (PMC1925077; doi:10.1186/1471-2458-7-127)
Supplement: Additional File 1 — Median survival (months) for four cohorts of AIDS cases at different dates of analysis according to the Kaplan-Meier estimator, December 1996 – December 2001. This table gives the estimate of median survival according to the Kaplan-Meier estimator for four cohorts for different dates of analysis in order to illustrate when a median survival estimate would be observed using traditional methods. [file 1471-2458-7-127-S1.doc]

Additional File 1. Median survival (months) for four cohorts of AIDS cases at different dates of analysis according to the Kaplan-Meier estimator, December 1996-December 2001.

|  |  | **Cohort** | | | | | | | | |
| --- | --- | --- | --- | --- | --- | --- | --- | --- | --- | --- |
|  | **Date of Analysis** | **933** | | **934** | | | **941** | **942** | | |
|  | 12/31/1996 | * | | * | | | * | * | | |
|  | 03/31/1997 | * | | * | | | * | * | | |
|  | 06/30/1997 | 45 | | * | | | * | * | | |
|  | 09/30/1997 | 40 | | * | | | * | * | | |
|  | 12/31/1997 | 40 | | * | | | * | * | | |
|  | 03/31/1998 | 39 | | * | | | * | * | | |
|  | 06/30/1998 | 38 | | 52 | | | * | * | | |
|  | 09/30/1998 | 38 | | 52 | | | * | * | | |
|  | 12/31/1998 | 37 | | 50 | | | * | * | | |
|  | 03/31/1999 | 37 | | 50 | | | * | * | | |
|  | 06/30/1999 | 37 | | 49 | | | * | * | | |
|  | 09/30/1999 | 36 | | 45 | | | 62 | * | | |
|  | 12/31/1999 | 36 | | 45 | | | 60 | * | | |
|  | 03/31/2000 | 36 | | 45 | | | 59 | * | | |
|  | 06/30/2000 | 36 | | 45 | | | 57 | * | | |
|  | 09/30/2000 | 34 | | 40 | | | 49 | * | | |
|  | 12/31/2000 | 34 | | 39 | | | 48 | * | | |
|  | 03/31/2001 | 34 | | 39 | | | 48 | * | | |
|  | 06/30/2001 | 34 | | 39 | | | 48 | 82 | | |
|  | 09/30/2001 | 34 | | 39 | | | 48 | 81 | | |
|  | 12/31/2001 | 34 | | 39 | | | 48 | 80 | | |
|  | | |  | |  |  | | |  |  |

Legend:

* denotes that the median could not be estimated from the data.
