# Supplementary material for: Early prediction of median survival among a large AIDS surveillance cohort
Source: BMC Public Health. 2007 Jun 27;7:127. doi: 10.1186/1471-2458-7-127 (PMC1925077; doi:10.1186/1471-2458-7-127)
Supplement: Additional File 2 — Comparison of Prediction Methodology with Traditional Methods (Kaplan-Meier Estimator). This table gives the earliest date the true median survival estimate could be observed and estimated using traditional methods (the Kaplan-Meier estimate), as well as the date any median survival estimate could be observed and estimated using the traditional approach. [file 1471-2458-7-127-S2.doc]

Additional File 2. Comparison of Prediction Methodology with Traditional Methods (Kaplan-Meier Estimator)

| | Cohort | Estimate from Our Method of Prediction  (A)† | | True Median Estimate (According to the IPCW method)  (B)‡ | | Earliest Date the True Median could be Estimated using K-M  (C)٭ | | Earliest Date a Median could be Estimated using K-M  (D)٭٭ | | | --- | --- | --- | --- | --- | --- | --- | --- | --- | | Date of Data | Predicted Median (months) | Date of Data | Median | Date of Data | Median | Date of Data | Predicted Median (months) | | 933 | 12/31/1996 | 34 | 12/31/2001 | 32 | >12/31/2001 | NA | 6/30/1997 | 45 | | 934 | 12/31/1996 | 34 | 12/31/2001 | 40 | 9/30/2000 | 40 | 5/31/1998 | 52 | | 941 | 12/31/1996 | 31 | 12/31/2001 | 46 | >12/31/2001 | NA | 7/31/1999 | 64 | | 942 | 12/31/1996 | 29 | 12/31/2001 | 80 | 11/30/2001 | 80 | 6/30/2001 | 82 |   IPCW, Inverse Probability of Censoring Weighted Estimator  K-M, Kaplan-Meier Estimator  NA, not available; median survival cannot be estimated using the latest available data (data as of December 31, 2001).  † Estimate from Our Method of Prediction (Column A): The predicted median survival for each of the four cohorts is estimated using data as of December 31, 1996.  ‡ True Median Estimate (According to the IPCW method) (Column B): Using the IPCW estimator with data as of December 31, 2001,   these two columns show that the median survival is as of this date (the “true” median survival estimate for each of the cohorts).  ٭ Earliest Date the True Median could be Estimated using the K-M estimator (Column C): If we did not have our method of prediction,   these two columns show when the true median could be estimated using the K-M estimator.  ٭٭Earliest Date a Median could be Estimated using the K-M estimator (Column D): If we did not have our method of prediction,   these two columns show what the first median estimate was (according to the K-M estimator) and when this median could be estimated. |  |  |  |  |  |  |  |  |
| --- | --- | --- | --- | --- | --- | --- | --- | --- | --- | --- | --- | --- | --- | --- | --- | --- | --- | --- | --- | --- | --- | --- | --- | --- | --- | --- | --- | --- | --- | --- | --- | --- | --- | --- | --- | --- | --- | --- | --- | --- | --- | --- | --- | --- | --- | --- | --- | --- | --- | --- | --- | --- | --- | --- | --- | --- | --- | --- | --- | --- | --- |
