# Supplementary material for: Early prediction of median survival among a large AIDS surveillance cohort
Source: BMC Public Health. 2007 Jun 27;7:127. doi: 10.1186/1471-2458-7-127 (PMC1925077; doi:10.1186/1471-2458-7-127)
Supplement: Additional File 3 — Predicted 0.50 Quantile versus "True" 0.50 Quantile according to the Inverse Probability of Censoring Weighted Estimate of Survival, 1983–1992. This table gives predicted and true 0.50 quantile estimates of survival using the methodology presented in the paper using a dataset as of December 31, 1992 and comparing it with results as of December 31, 1997 (assumed to be the "truth"). [file 1471-2458-7-127-S3.doc]

Additional File 3. Predicted 0.50 Quantile versus “True” 0.50 Quantile According to the Inverse Probability of Censoring Weighted Estimate of Survival, 1983-1992

|  |  | Data as of  December 31, 1992 | | |  | Data as of  December 31, 1997 | |  |
| --- | --- | --- | --- | --- | --- | --- | --- | --- |
|  |  |  | Observed Quantiles | |  |  |  |  |
| Cohort | n | largest follow-up time (months) | 0.15  (A) | 0.50 (B) | Predicted  0.50†  (C) | largest follow-up time (months) | "True" 0.50‡  (D) | Difference  (C – D) |
|  |  |  |  |  |  |  |  |  |
| 831 | 136 | 119 | 2 | 12 | 15 | 179 | 10 | 5 |
| 832 | 152 | 116 | 2 | 10 | 15 | 176 | 11 | 4 |
| 833 | 199 | 113 | 1 | 11 | 13 | 173 | 11 | 2 |
| 834 | 204 | 110 | 1 | 10 | 13 | 170 | 9 | 4 |
| 841 | 270 | 107 | 1 | 9 | 13 | 167 | 10 | 3 |
| 842 | 307 | 104 | 1 | 11 | 13 | 164 | 11 | 2 |
| 843 | 388 | 101 | 1 | 12 | 13 | 161 | 11 | 2 |
| 844 | 408 | 98 | 1 | 10 | 13 | 158 | 10 | 3 |
| 851 | 516 | 95 | 1 | 10 | 13 | 155 | 10 | 3 |
| 852 | 610 | 92 | 1 | 9 | 13 | 152 | 10 | 3 |
| 853 | 702 | 89 | 1 | 11 | 13 | 149 | 11 | 2 |
| 854 | 672 | 86 | 1 | 10 | 13 | 146 | 10 | 3 |
| 861 | 857 | 83 | 1 | 10 | 13 | 143 | 10 | 3 |
| 862 | 931 | 80 | 1 | 10 | 13 | 140 | 10 | 3 |
| 863 | 1083 | 77 | 1 | 10 | 13 | 137 | 11 | 2 |
| 864 | 1079 | 74 | 1 | 17 | 13 | 134 | 14 | -1 |
| 871 | 1285 | 71 | 1 | 15 | 13 | 131 | 14 | -1 |
| 872 | 1424 | 68 | 1 | 14 | 13 | 128 | 15 | -2 |
| 873 | 1424 | 65 | 1 | 16 | 13 | 125 | 16 | -3 |
| 874 | 1377 | 62 | 2 | 17 | 15 | 122 | 17 | -2 |
| 881 | 1577 | 59 | 1 | 17 | 13 | 119 | 17 | -4 |
| 882 | 1569 | 56 | 2 | 18 | 15 | 116 | 18 | -3 |
| 883 | 1658 | 53 | 2 | 17 | 15 | 113 | 18 | -3 |
| 884 | 1591 | 50 | 1 | 18 | 13 | 110 | 17 | -4 |
| 891 | 1771 | 47 | 2 | 17 | 15 | 107 | 17 | -2 |
| 892 | 1964 | 44 | 2 | 18 | 15 | 104 | 17 | -2 |
| 893 | 1812 | 41 | 2 | 18 | 15 | 101 | 17 | -2 |
| 894 | 1740 | 38 | 1 | 18 | 13 | 98 | 16 | -3 |

**Additional File 3 continued. Predicted 0.50 Quantile versus “True” 0.50 Quantile According to the Inverse Probability of Censoring Weighted Estimate of Survival, 1983-1992**

|  |  | Data as of  December 31, 1992 | | |  | Data as of  December 31, 1997 | |  |
| --- | --- | --- | --- | --- | --- | --- | --- | --- |
|  |  |  | Observed Quantiles | |  |  |  |  |
| Cohort | n | largest follow-up time (months) | 0.15  (A) | 0.50 (B) | Predicted  0.50†  (C) | largest follow-up time (months) | "True" 0.50‡  (D) | Difference  (C – D) |
|  |  |  |  |  |  |  |  |  |
| 901 | 1869 | 35 | 2 | 20 | 15 | 95 | 17 | -2 |
| 902 | 1883 | 32 | 2 | 19 | 15 | 92 | 18 | -3 |
| 903 | 1904 | 29 | 3 | 16 | 17 | 89 | 16 | 1 |
| 904 | 1670 | 26 | 2 | 15 | 15 | 86 | 15 | 0 |
| 911 | 1932 | 23 | 4 | 17 | 19 | 83 | 16 | 3 |
| 912 | 1809 | 20 | 3 | 16 | 17 | 80 | 16 | 1 |
| 913 | 1909 | 17 | 3 | 15 | 17 | 77 | 16 | 1 |
| 914 | 1746 | 14 | 2 | 11 | 15 | 74 | 15 | 0 |
| 921 | 1589 | 11 | 2 | * | 15 | 71 | 16 | -1 |
| 922 | 1246 | 8 | 3 | * | 17 | 68 | 16 | 1 |
| 923 | 918 | 5 | * | * | * | 65 | 14 |  |
| 924 | 415 | 2 | * | * | * | 62 | 16 |  |

Legend:

* denotes that the observed quantile could not be estimated from the data.

† The predicted 0.50 quantile is based on HARS data as of December 31, 1992.

‡ The true 0.50 quantile is based on HARS data as of December 31, 1997.
